# Prognostic value of systemic immune-inflammation index for patients undergoing radical prostatectomy: a systematic review and meta-analysis

**DOI:** 10.3389/fimmu.2025.1465971

**Published:** 2025-02-04

**Authors:** Zhan Chen, Yao Zhang, Wei Chen

**Affiliations:** ^1^ Department of Urology, Cixilntegrated Traditional Chinese and Western Medicine Medical, Ningbo, Zhejiang, China; ^2^ Department of Urology, Ningbo Yinzhou No.2 Hospital, Ningbo, Zhejiang, China

**Keywords:** systemic immune-inflammation index, prostate cancer, prostatectomy, meta-analysis, SII

## Abstract

**Objective:**

The prognostic value of the systemic immune-inflammation index (SII) for prostate cancer (PCa) patients receiving different treatments remains unclear. This research examined the relevance of SII in individuals undergoing radical prostatectomy (RP).

**Methods:**

PubMed, Embase, Web of Science, Cochrane, Wanfang, and China National Knowledge Infrastructure (CNKI) dat3 abases were used to search literature up to May 2024. The quality was evaluated with Newcastle-Ottawa Scale. Outcomes examined were associations between SII and overall survival (OS), biochemical recurrence-free survival (BFS), and cancer-specific survival (CSS). Pooled analysis, Egger’s test, and sensitivity analysis were conducted using Review Manager 5.4.1 and Stata 15.1. The GRADE system was employed to evaluate and grade the evidence for each outcome. Subgroup analyses were performed for outcomes with significant heterogeneity to evaluate the possible confounders, if data were sufficient.

**Results:**

Out of 101 identified studies, eight studies involving 8,267 individuals were included. Patients with higher SII had shorter overall survival (HR: 1.89; 95% CI: 1.31-2.71; P = 0.0006), biochemical recurrence-free survival (HR: 1.55; 95% CI: 1.08-2.22; P = 0.02), and cancer-specific survival (HR: 3.63; 95% CI: 1.66-7.94; P = 0.001). The evidence for OS and CSS was rated very low-quality due to serious heterogeneity and/or imprecision. The prognostic value of SII for BFS was rated as low-quality evidence, given no serious risk observed. Subgroup analysis showed that, except for the subgroup aged >65 years (HR: 3.70; 95%CI: 0.91, 15.06, *P*=0.07), the prognostic value of SII for OS was not significant, but the prognostic value of SII for OS in other subgroups was still significant.

**Conclusions:**

High SII was linked to shorter OS, BFS, and CSS in patients undergoing RP. However, the quality of the evidence provided by this study was low.

**Systematic Review Registration:**

https://www.crd.york.ac.uk/PROSPERO/, identifier CRD42024558431.

## 1 Introduction

Prostate cancer (PCa), one of the most common malignancies in middle-aged and elderly men, ranks fourth among global malignancies and second among male malignancies (1). Recent statistical data indicate an annual increase of 28,300 PCa cases in the United States, where it is the most prevalent male tumor, with approximately 34,700 deaths annually (2). Currently, radiotherapy and radical prostatectomy (RP) is the most effective treatment for localized PCa (3). Studies report that biochemical recurrence (BCR) occurs in about 27%-53% of patients with clinically localized PCa after RP (4), greatly affecting patient prognosis. BCR often indicates that patients will develop local recurrence or distant metastasis. Early identification of patients prone to BCR through certain biomarkers is essential, allowing for timely interventions such as hormonal therapy to improve prognosis, survival, and life quality (5).

The impact of immunoinflammatory cells on tumor progression and patient outcomes has been documented across various solid tumors. Research indicates that the neutrophil-to-lymphocyte ratio (NLR) and the platelet-to-lymphocyte ratio (PLR) are significantly associated with the prognosis of digestive system malignancies (6–8), breast cancer (9), lung cancer (10), and kidney cancer (11). However, NLR and PLR only account for two types of inflammatory cells, often limiting their prognostic accuracy. The systemic immune-inflammation index (SII), calculated as neutrophil count multiplied by platelet count and divided by lymphocyte count, offers a more comprehensive inflammatory marker incorporating lymphocyte (L), neutrophil (N), and platelet (P) counts. Recently, SII has been employed to assess the balance between preoperative inflammation and immune status, proving to be a significant prognostic indicator for survival and recurrence in various cancers, such as hepatocellular carcinoma (12), germ cell tumors (13), and bladder cancer (14), potentially outperforming other inflammatory markers.

Several studies have shown that SII levels can be used as an important indicator to predict the prognosis of PCa patients (15). PCa patients with high SII levels usually have higher clinical stages and pathological grades, and are more likely to have lymph node metastasis and bone metastasis. The prognosis of these patients is usually poor. Therefore, SII levels can be used as an important reference for judging the prognosis of PCa patients (16). The application value of SII in the prognosis assessment of PCa is not only reflected in its independent predictive ability, but also in its combined application with other traditional detection methods. Studies have shown that the diagnostic significance of SII levels alone is limited, but combined with traditional detection methods such as digital rectal examination (DRE) and prostate-specific antigen (PSA) can improve the diagnostic efficiency (17). This multi-factor joint prediction model has shown good value in the diagnosis and prognosis assessment of PCa.

Meng et al. (18) performed a meta-analysis, revealing that elevated SII might be linked to poorer OS and PFS. However, this analysis did not differentiate between PCa patients who underwent RP and those receiving non-surgical combined treatments, making it unclear if SII’s prognostic value varies with different treatment modalities. Additionally, recent studies present inconsistent conclusions and lack robust evidence-based medical validation (19, 20). Consequently, this study aims to conduct a meta-analysis to explore the relationship between SII levels and prognosis in PCa patients undergoing RP, with the objective of systematically evaluating SII’s prognostic significance in this particular patient group.

## 2 Methods

### 2.1 Literature search

The PRISMA 2020 guidelines were followed (21) and this meta-analysis was registered prospectively in PROSPERO (CRD42024558431). PubMed, Embase, Web of Science, Cochrane, Wanfang, and China National Knowledge Infrastructure (CNKI) databases were utilized for searching literature from their inception to May 2024, focusing on studies evaluating the prognostic value of SII in patients undergoing RP. Search terms included “Prostatectomy”, “systemic immune-inflammation index”, and “SII”. The detailed search strategy was: ((“Prostatectomy”[Mesh]) OR (Prostatectomies OR Retropubic Prostatectomies OR Retropubic Prostatectomy OR Suprapubic Prostatectomies OR Suprapubic Prostatectomy)) AND (“systemic immune-inflammation index” OR SII). Additionally, reference lists of included studies were manually screened. Two authors independently retrieved and assessed eligible articles, resolving discrepancies through discussion. Search details are provided in **Supplementary Table S1**.

### 2.2 Inclusion and exclusion criteria

Studies were included based on the following criteria (1): randomized controlled trials, cohort studies, or case-control designs; (2) participants who underwent RP; (3) investigation of the prognostic significance of SII in patients undergoing RP; (4) evaluation of at least one survival outcome, such as overall survival (OS), free survival (BFS), or cancer-specific survival (CSS); and (5) provision of adequate data to calculate hazard ratios (HR) with 95% confidence intervals (95% CIs). Exclusion criteria included study protocols, unpublished studies, non-original articles (e.g., letters, comments, abstracts, corrections, and replies), studies lacking sufficient data, and reviews.

### 2.3 Data abstraction

Data from the selected studies were independently extracted by two authors (ZC and YZ), with any disagreements resolved by a third author (WC). The collected data included the first author’s name, publication year, study duration, geographic location, study design, population characteristics, sample size, age, body mass index (BMI), tumor size, prostate-specific antigen (PSA) levels, follow-up duration, SII cut-off values, overall survival (OS), cancer-specific survival (CSS), and biochemical recurrence-free survival (BFS). When data were incomplete, corresponding authors were contacted for additional information.

### 2.4 Quality evaluation

The quality of the cohort studies included in this review was evaluated using the Newcastle-Ottawa Scale (NOS) (22). Studies that scored between 7 and 9 points were classified as high quality (23), while those scoring below 6 were excluded from the quantitative analysis. Two authors (ZC and YZ) independently performed the quality assessment of all included studies.

### 2.5 Statistical analysis

The meta-analysis utilized Review Manager 5.4.1 to synthesize survival data with hazard ratios (HRs), presenting effect sizes along with 95% confidence intervals (CIs). Heterogeneity among studies was assessed using the chi-squared (χ2) test (Cochran’s Q) and the inconsistency index (I^2^) (24), with significant heterogeneity indicated by a χ2 P value < 0.1 or an I^2^ > 50%. A random-effects model was employed to calculate the pooled HR for each outcome. Sensitivity analyses were performed to assess the impact of each included study on the pooled HR for all outcomes. Publication bias was evaluated using funnel plots created in Review Manager 5.4.1 and Egger’s regression tests (25) conducted in Stata 15.1 (Stata Corp, College Station, Texas, USA), with a P value < 0.05 indicating statistically significant publication bias. The quality of evidence for each outcome was assessed using the GRADE approach and categorized as “high,” “moderate,” “low,” or “very low” to draw conclusions (26). In addition, subgroup analyses were performed for outcomes with significant heterogeneity to evaluate the possible confounders, if data were sufficient.

## 3 Results

### 3.1 Literature retrieval, study characteristics, and baseline

**Figure 1:**
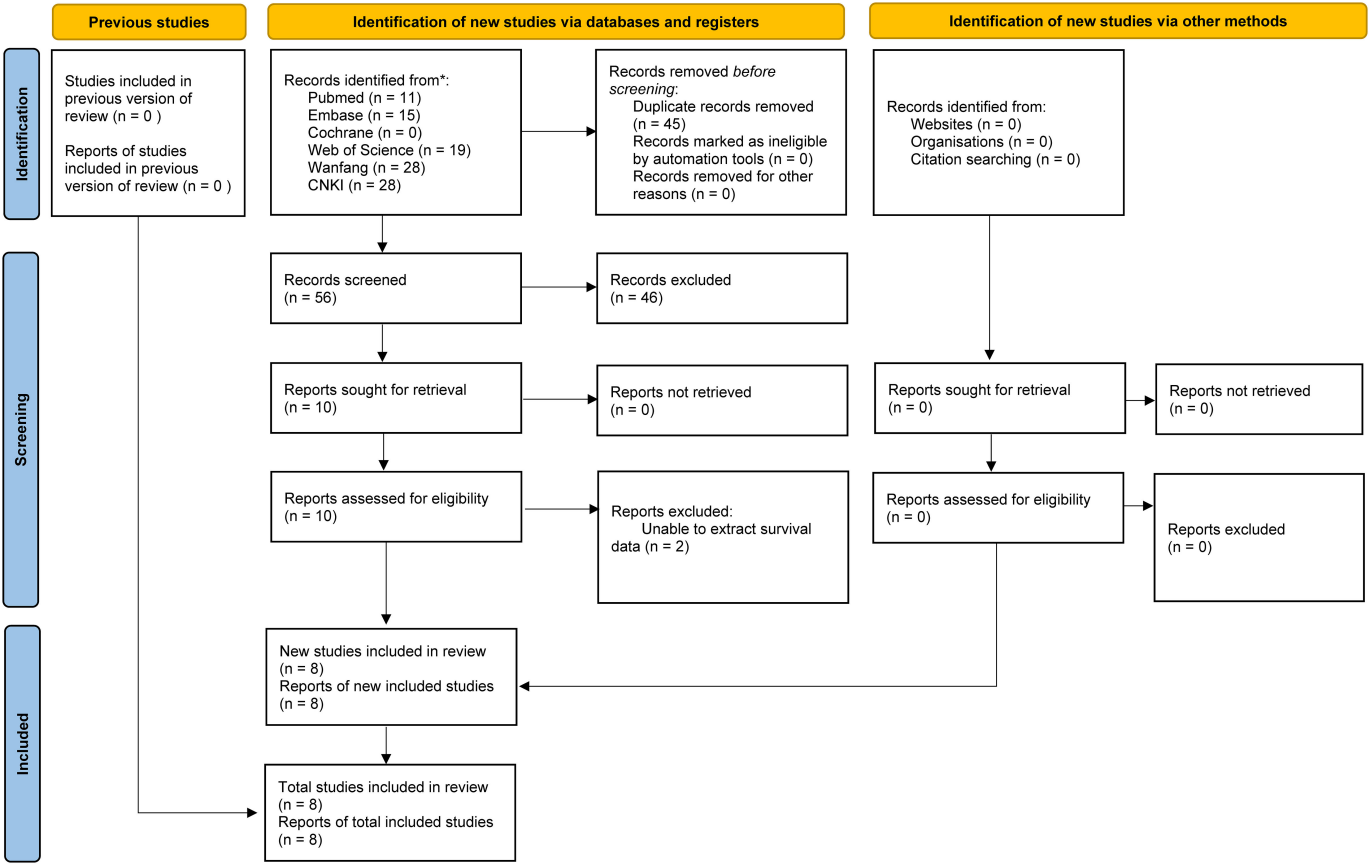
Flowchart of the systematic search and selection process.

Literature retrieval and selection process exhibits in **Figure 1**. A systematic search across various databases identified 101 related studies: PubMed (n = 11), Embase (n = 15), Web of Science (n = 19), Cochrane (n = 0), Wanfang (n = 28), and CNKI (n = 28). After removing duplicates, 56 titles and abstracts were screened. Ultimately, 8 cohort studies (8,267 patients) reserved (19, 20, 26–31). In all studies, SII was measured and calculated before surgery. **Table 1** provides detailed information on the characteristics and quality assessments of each included cohort study. Supplementary information for all included articles is provided in **Supplementary Table S2**.

**Table 1:** Characteristics and quality evaluation of each eligible cohort study.

Study	Study period	Country	Study design	No. of patients	Mean/median age	Mean/median BMI	cT stage	SII threshold	NOS score
Bailey-Whyte 2023	2005-2015	USA	Retrospective cohort	680	63 (median)	28.2 (mean)	T1-T4	430.8	7
Li 2023	2017-2022	China	Retrospective cohort	403	68.39 (mean)	NA	T2-T4	731	9
Rajwa 2021a	2000-2011	Multicenter	Retrospective cohort	6039	61 (median)	28 (median)	T3-T4	620	7
Rajwa 2021b	2007-2015	Multicenter	Retrospective cohort	81	69 (median)	24 (median)	T1-T4	730	8
Shi 2023	2016-2019	China	Retrospective cohort	150	68.4 (mean)	23 (mean)	T1-T4	402.48	8
Wu 2023	2016-2021	China	Retrospective cohort	290	67.53 (mean)	NA	T2-T4	NA	7
Yao 2022	2012-2019	China	Retrospective cohort	203	NA	NA	T2-T4	517.61	8
Zapala 2022	2012-2018	Poland	Retrospective cohort	421	65 (median)	NA	T1-T4	900	9

NA, Not available.

### 3.2 OS

**Figure 2:**
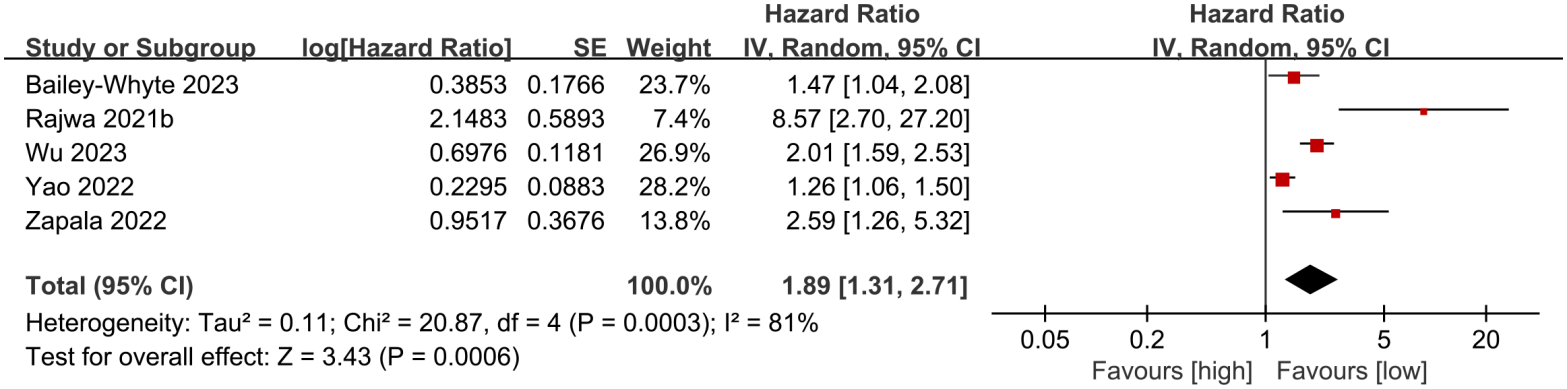
Forest plots of OS.

Five cohort studies (19, 27, 29–31) were included in the meta-analysis examining overall survival (OS). Results indicated that patients with high SII experienced significantly shorter OS compared to those with low SII (HR: 1.89; 95% CI: 1.31, 2.71; P = 0.0006). Significant heterogeneity showed among the studies (I^2^ = 81%, P = 0.0003) (**Figure 2**).

### 3.3 BFS

**Figure 3:**
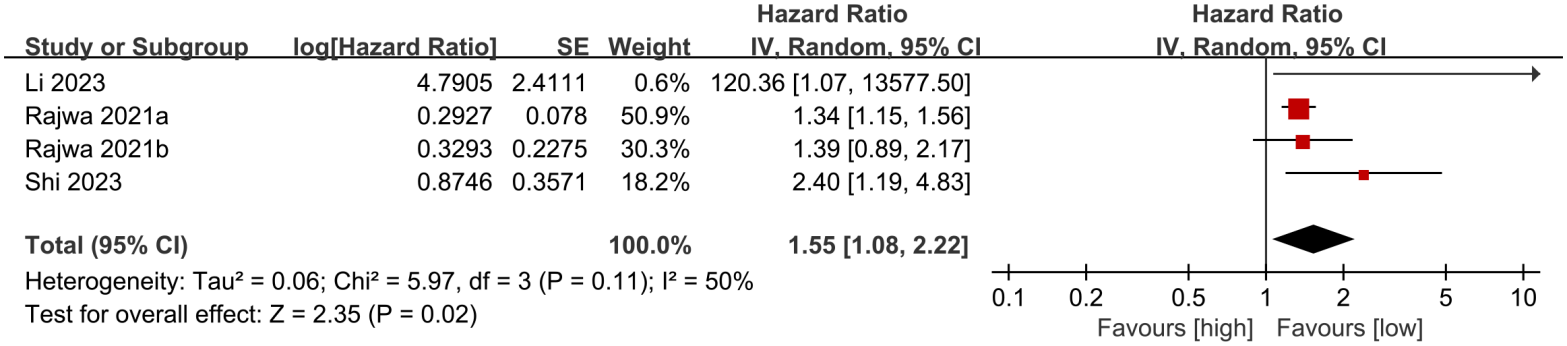
Forest plots of BFS.

Four cohort studies (20, 26–28) were incorporated into the meta-analysis of biochemical recurrence-free survival (BFS). The findings showed that patients with elevated SII had significantly shorter BFS compared to those with lower SII (HR: 1.55; 95% CI: 1.08, 2.22; P = 0.02). There was no significant heterogeneity (I^2^ = 50%, P = 0.11) (**Figure 3**).

### 3.4 CSS

**Figure 4:**
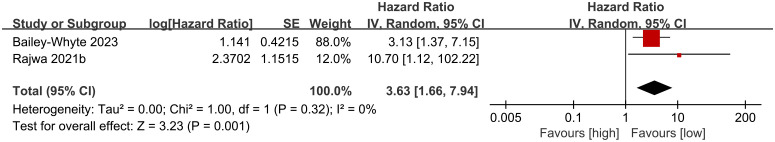
Forest plots of CSS.

Two cohort studies (19, 27) were part of the meta-analysis for cancer-specific survival (CSS). The findings indicated that patients with elevated SII had notably shorter CSS compared to those with lower SII (HR: 3.63; 95% CI: 1.66, 7.94; P = 0.001). There was no significant heterogeneity (I^2^ = 0%, P = 0.32) (**Figure 4**).

### 3.5 Publication bias and sensitivity analysis

**Figure 5:**
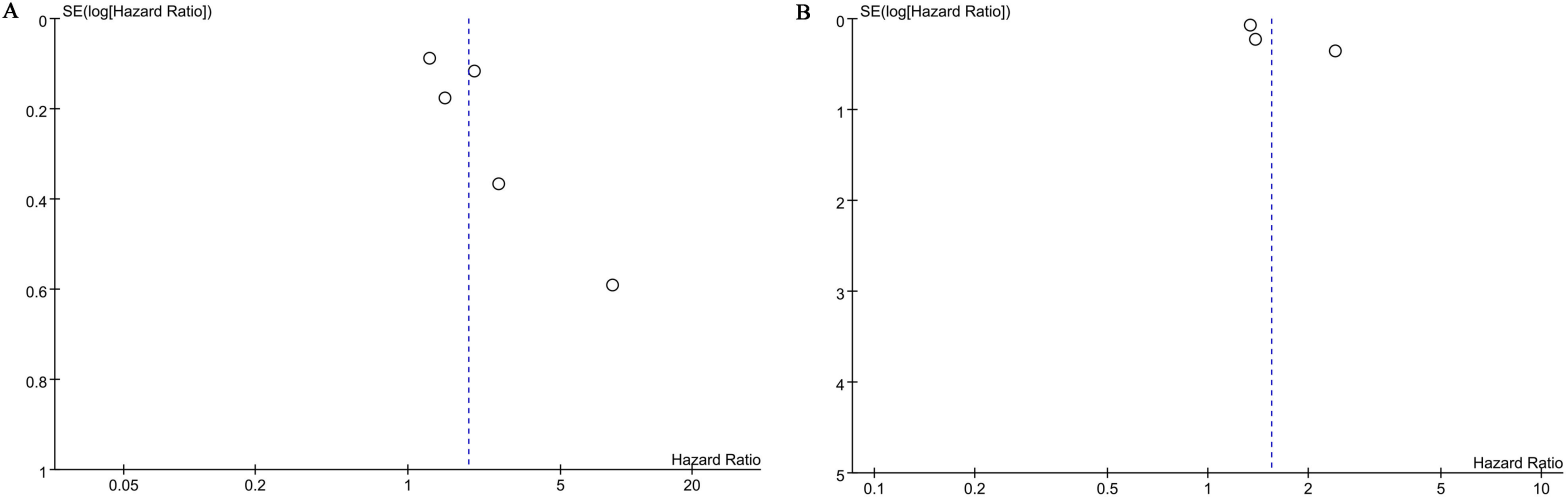
Funnel plots of OS **(A)** and BFS **(B)**.

**Figure 6:**
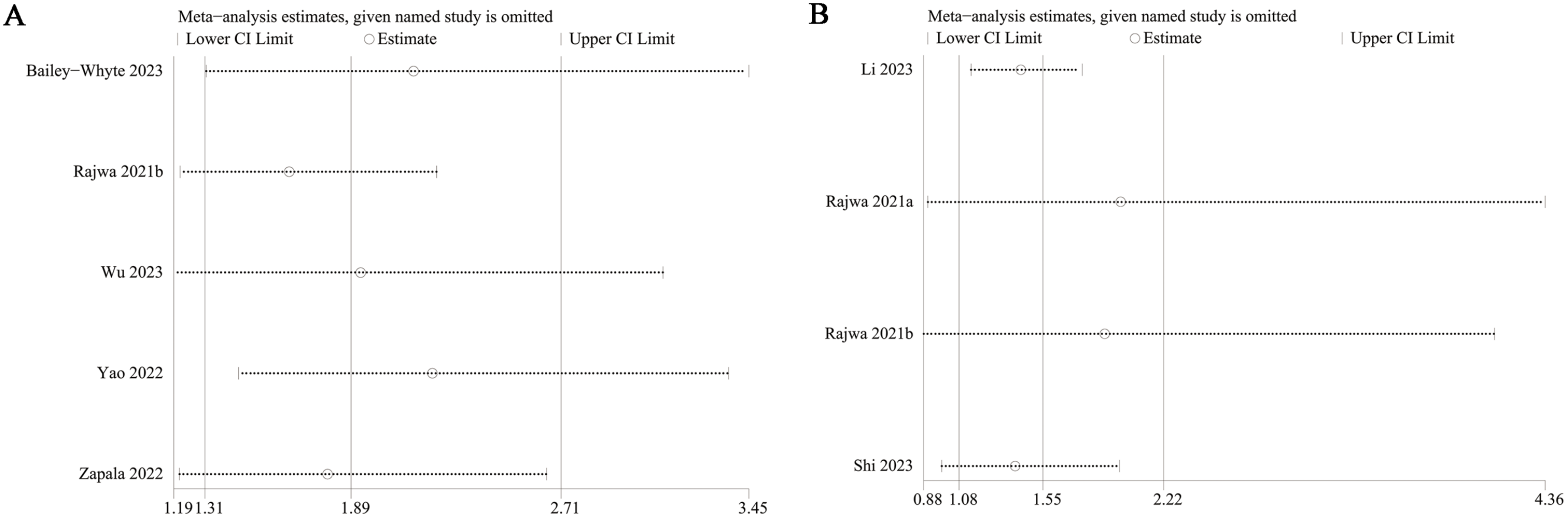
Sensitivity analysis of OS **(A)** and BFS **(B)**.

</b>Publication bias for overall survival (OS) and biochemical recurrence-free survival (BFS) was evaluated using both funnel plots and Egger’s regression tests. No statistical (Egger’s test) or visual (funnel plots) evidence of publication bias was observed for OS (Egger’s test P = 0.138) (**Figure 5A**) or BFS (Egger’s test P = 0.087) (**Figure 5B**). Sensitivity analyses were performed for OS and BFS to assess the effect of each cohort study on the pooled hazard ratio (HR) by sequentially excluding individual studies. For OS, the pooled HR remained consistent after excluding each study one by one (**Figure 6A**). However, for BFS, the removal of data from Rajwa 2021a (20), Rajwa 2021b (27), or Shi 2023 (28) altered the significance of the difference (**Figure 6B**).

### 3.6 GRADE rating

The evidence quality for the prognostic significance of SII was rated as very low for overall survival (OS) and cancer-specific survival (CSS) due to significant heterogeneity and/or imprecision. For biochemical recurrence-free survival (BFS), the quality of evidence was deemed low, given the absence of a serious risk of bias (**Table 2**).

**Table 2:** GRADE rating of each outcome.

No. of studies	Outcomes	HR	95%CI	*I* ^2^; P value	Risk of bias	Inconsistency	Indirectness	Imprecision	Publication bias	Plausible confounding	Magnitude of effect	Dose-response gradient	GRADE
5	OS	1.89	1.31, 2.71	81%; P =0.0003	No serious risk	Serious inconsistency	No serious indirectness	No serious imprecision	Undetected	Would not reduce effect	No	No	Very low
4	BFS	1.55	1.08, 2.22	50%; P=0.11	No serious risk	No serious inconsistency	No serious indirectness	No serious imprecision	Undetected	Would not reduce effect	No	No	Low
2	CSS	3.63	1.66, 7.94	0%; P=0.32	No serious risk	No serious inconsistency	No serious indirectness	Serious imprecision	NA	Would not reduce effect	No	No	Very low

### 3.7 Subgroup analysis

This study conducted a subgroup analysis of OS based on sample size, region, age, and SII cutoff value. The results showed that, except for the subgroup aged >65 years (HR: 3.70; 95%CI: 0.91, 15.06, *P*=0.07), the prognostic value of SII for OS was not significant, but the prognostic value of SII for OS in other subgroups was still significant (**Table 3**). In addition, subgroup analysis suggested that sample size, age, and SII cutoff value were the main reasons for the significant heterogeneity in OS.

**Table 3:** Subgroup analysis of OS.

Subgroup	OS			
	Study	HR [95%CI]	*P* value	*I* ^2^
Total	5	1.89 [1.31-2.71]	0.0006	81%
Sample size				
≥300	2	1.78 [1.05-3.01]	0.03	48%
<300	3	2.04 [1.18-3.54]	0.01	89%
Region				
China	2	1.58 [1.00-2.50]	0.05	90%
USA	1	1.47 [1.04-2.08]	0.03	/
Poland	1	2.59 [1.26-5.32]	0.01	/
Multicenter	1	8.57 [2.70-27.20]	0.0003	/
Mean/median age				
>65y	2	3.70 [0.91-15.06]	0.07	83%
≤65y	2	1.78 [1.05-3.01]	0.03	48%
SII cut-off				
≥600	2	4.31 [1.35-13.75]	0.01	66%
<600	2	1.30 [1.11-1.52]	0.001	0%

## 4 Discussion

The systemic immune-inflammation index (SII), which reflects the balance between autoimmunity and inflammation, is calculated from neutrophil, platelet, and lymphocyte levels. SII can monitor immune status and has shown better prognostic reliability for prognosis of patients with lung cancer (32). Presently, SII is mainly used in prognostic assessments for liver and colorectal cancers. A 2016 study first linked SII with renal cell carcinoma, suggesting it as a prognostic marker for metastatic renal cell carcinoma patients, with a cutoff value of 535.0 (33). A recent study suggested that SII might be an effective prognostic marker for patients with metastatic castration-resistant PCa receiving docetaxel treatment (34). However, the use of biomarkers like SII for prognosis in cancer patients remains controversial. Li et al. (35) investigated the prognostic and clinical significance of preoperative SII in bladder cancer patients, finding that elevated preoperative SII was significantly associated with poor survival outcomes and adverse pathological features, making SII an independent predictor of postoperative prognosis in bladder cancer patients. In contrast, Rajwa et al. (27), in a multicenter retrospective study using logistic and Cox regression analyses, evaluated the prognostic value of preoperative SII and found that it did not predict biochemical recurrence-free survival (BFS) in patients undergoing RP.

Our research demonstrated that SII is a significant predictor of overall survival (OS), biochemical recurrence-free survival (BFS), and cancer-specific survival (CSS) in patients undergoing RP. These findings align with a previously published meta-analysis. Meng et al. (18) conducted a meta-analysis showing that high SII was associated with worse OS in PCa patients (HR = 1.44, 95% CI 1.23-1.69, p < 0.001). They also found a correlation between increased SII and poorer progression-free survival (PFS) (HR = 1.80, 95% CI 1.27-2.56, p = 0.001). Building on their work, our meta-analysis further explored the prognostic value of SII in patients undergoing RP, specifically focusing on OS, BFS, and CSS. Additionally, we applied the GRADE approach to evaluate the quality of evidence, finding that SII had the highest level of evidence for predicting BFS. However, sensitivity analyses indicated significant instability in the prognostic value of SII for BFS. Consequently, further prospective studies are needed to confirm whether SII can reliably predict the long-term prognosis of patients undergoing RP.

As the primary components of peripheral white blood cells, neutrophils are produced at a rate exceeding 10¹¹ cells per day and play a crucial role in the immune response (36–38). Recent studies have shown that tumors can disrupt normal neutrophil homeostasis. Tumor cells secrete pro-inflammatory cytokines that attract neutrophils to the cancer site and induce their conversion into pro-tumor neutrophils, thereby promoting tumor metastasis, proliferation, and immunosuppression (39). Neutrophil-derived inflammatory mediators can also modulate the tissue and tumor microenvironment (TME), fostering tumor development, angiogenesis, progression, and metastasis (40–43). Lymphocytopenia, commonly observed in patients with advanced tumors, leads to an immunosuppressive state (44). Lymphocytes produce inhibitory cytokines that induce programmed cell death and regulate tumor cells (45, 46). Consequently, a reduction in lymphocyte count may result in a weakened immune response against malignant tumors, diminishing the inhibitory effect on tumor proliferation and enabling rapid tumor cell growth (47). Platelets play a critical role in the progression of many malignancies, contributing to local tumor growth, spread, and metastasis (48–51). At the tumor site, platelets can be activated by tumor cell-secreted thrombin and tissue factor expression, forming a physical barrier of platelet-fibrin mesh that protects cancer cells from potential natural killer (NK) cell attack (52). Additionally, activated platelets release various cytokines that promote tumor growth and angiogenesis (53). Thus, platelet count may serve as an indicator of disease progression in cancer patients (54).

To date, PSA is still the most widely used serum marker in clinical diagnosis. However, due to the fact that the specificity of PSA is only 59.2%, PSA is often easily affected by other factors. For example, elevated PSA levels are found in the blood of patients with benign prostate diseases (prostatic hyperplasia, prostatitis) (55). In addition, when taking some drugs (5α-reductase inhibitors), the side effects of the drugs can also cause a decrease in the patient’s serum PSA level (56). At the same time, PSA cannot accurately predict the prognosis of patients after PCa surgery. Therefore, a relatively easy-to-obtain clinical indicator is needed to predict the postoperative situation of PCa and better monitor the long-term prognosis of patients. The findings of this study suggest that high SII is significantly associated with shorter OS, BFS and CSS after PCa surgery, and can be used as an predictor of PCa surgery. This finding provides a cheap and sensitive detection method for predicting the long-term prognosis after PCa surgery, which helps to accurately identify high-risk individuals and guide clinical treatment.

Studies have shown that SII is an indicator that can be measured in many chronic diseases, including cardiovascular disease, cancer, and autoimmune diseases. For example, in the field of coronary heart disease, SII has been confirmed by many studies to be an independent predictor of prognosis. For example, a study on patients with coronary heart disease found that SII levels were significantly correlated with patients’ survival rates, and the cumulative survival rate of patients in the high SII group was significantly lower than that in the low SII group (57). In addition, SII also showed higher predictive ability compared with other traditional biomarkers such as N-terminal pro-B-type natriuretic peptide (NT-proBNP) and soluble growth-stimulating gene expression protein 2 (sST2) (58). These studies have shown that SII can be used as an important tool for prognostic assessment in patients with coronary heart disease. In addition to coronary heart disease and myocardial infarction, SII has also shown certain value in the prognostic assessment of other cardiovascular diseases such as arrhythmias, cardiomyopathy, and infective endocarditis. For example, SII levels are associated with the risk of death in patients with hypertrophic cardiomyopathy and can predict mid-term outcomes (59); high SII values ​​are independent predictors of high mortality in patients with infective endocarditis (60, 61).

This meta-analysis has several limitations that should be acknowledged. Firstly, it included only retrospective cohort studies, which are susceptible to potential confounders and uncontrolled risk of bias. Future large-sample prospective cohort studies with well-designed methodologies are necessary to address these limitations. Secondly, the studies included were conducted in Europe, Asia and America, lacking population data from other regions or countries, thereby making the generalizability of the findings to other regions uncertain. Furthermore, significant heterogeneity was observed in some outcomes. However, this study identified the main sources of heterogeneity through subgroup analysis. In addition, due to limited original data, this study only retrieved 11 relevant studies and did not include unpublished literature, which may have caused the possibility of missing data. Meanwhile, due to insufficient data, we were unable to conduct detailed subgroup analysis based on the pathological characteristics, surgical complications, treatment, and other information of PCa, which needs to be further studied to be resolved. At the same time, the results of individual meta-analyses and subgroup analyses included limited literature and there may be unavoidable small sample size effects, so caution should be exercised when interpreting their results. Finally, the prognostic value of SII for survival outcomes, such as PFS and DFS, could not be analyzed due to insufficient data. Despite these limitations, this study represents the most recent and comprehensive analysis of the prognostic value of SII in patients undergoing RP. The findings highlight the importance of monitoring changes in SII levels for the clinical management of patients following RP. In the future, the development of more robust prognostic models incorporating inflammatory markers such as SII is expected to improve the long-term prognosis and quality of life of PCa patients post-surgery.

## 5 Conclusion

Elevated SII was correlated with reduced OS, BFS, and CSS in patients who underwent RP. Because routine blood tests are inexpensive and straightforward, SII can be broadly employed to assess prognosis and establish risk prediction models for patients undergoing RP. However, the quality of the evidence provided by this study was low. Due to the limitations of retrospective studies, potential population selection bias, and heterogeneity, further large-scale, multi-center, prospective clinical studies are required to validate the association between SII and prognosis following RP.

## Data Availability

The original contributions presented in the study are included in the article/**Supplementary Material**. Further inquiries can be directed to the corresponding author.
