# Supplementary material for: Prognostic value of systemic immune-inflammation index for patients undergoing radical prostatectomy: a systematic review and meta-analysis
Source: Front Immunol. 2025 Feb 4;16:1465971. doi: 10.3389/fimmu.2025.1465971 (PMC11832501; doi:10.3389/fimmu.2025.1465971)
Supplement: Supplementary file 2 [file DataSheet2.docx]

Supplementary Table S1. Search strategy.

Pubmed-11

(("Prostatectomy"[Mesh]) OR (((((Prostatectomies) OR (Retropubic Prostatectomies)) OR (Retropubic Prostatectomy)) OR (Suprapubic Prostatectomies)) OR (Suprapubic Prostatectomy))) AND ((systemic immune-inflammation index) OR (SII))

Embase-15

((Prostatectomy or Prostatectomies or Retropubic prostatectomies or Retropubic prostatectomy or suprapubic prostatectomies or suprapubic prostatectomy) and sstemic immune inflammation index or Sll)).af.

Cochrane-0

((Prostatectomy or Prostatectomies or Retropubic prostatectomies or Retropubic prostatectomy or suprapubic prostatectomies or suprapubic prostatectomy) and sstemic immune inflammation index or Sll)).af.

WOS-19

((Prostatectomy) OR (((((Prostatectomies) OR (Retropubic Prostatectomies)) OR (Retropubic Prostatectomy)) OR (Suprapubic Prostatectomies)) OR (Suprapubic Prostatectomy))) AND ((systemic immune-inflammation index) OR (SII)) (Topic) and Preprint Citation Index (Exclude – Database)

Wanfang-28

全部:(前列腺切除) and 全部:(免疫炎症指数 OR SII)

CNKI-28

（篇关摘：前列腺切除(模糊)）AND（篇关摘：免疫炎症指数 + SII(模糊)）
