# Supplementary material for: Prognostic value of systemic immune-inflammation index for patients undergoing radical prostatectomy: a systematic review and meta-analysis
Source: Front Immunol. 2025 Feb 4;16:1465971. doi: 10.3389/fimmu.2025.1465971 (PMC11832501; doi:10.3389/fimmu.2025.1465971)
Supplement: Supplementary file 3 [file DataSheet3.docx]

| Supplementary Table S2. Supplementary information for all included articles. | | | | | |
| --- | --- | --- | --- | --- | --- |
| Study | Types of radical prostatectomy (RP) | Proportion of LN+ patients | Median OS | Median BFS | Median CSS |
|  |  |  |  |  |  |
| Bailey-Whyte 2023 | NA | NA | 5.9 years | NA | 5.85 years |
| Li 2023 | Laparoscopic and (56.9%) robotic assistance (43.1%) | NA | 15 months | NA | NA |
| Rajwa 2021a | NA | 1.9% | 44 moths | 20 months | NA |
| Rajwa 2021b | Salvage RP | 19% | 25.3 months | NA | NA |
| Shi 2023 | NA | NA | 46.0 months | NA | NA |
| Wu 2023 | Laparoscopic | 41.7% | NA | NA | NA |
| Yao 2022 | NA | 17.2% | NA | NA | NA |
| Zapala 2022 | NA | 7.4% | 69 months | NA | NA |
